# Vaccine Coadministration in Primary Care: A KAP Survey Among General Practitioners in Southern Italy

**DOI:** 10.3390/vaccines14090725

**Published:** 2026-08-22

**Authors:** Cesare De Virgilio Suglia, Maria Zamparella, Claudia Palmieri, Chiara Noviello, Pasquale Stefanizzi, Silvio Tafuri

**Affiliations:** 1 Interdisciplinary Department of Medicine (DIM), University of Bari Aldo Moro, 70121 Bari, Italypasquale.stefanizzi@uniba.it (P.S.); 2 SIMCCP—Società Italiana di Medicina di Comunità e Cure Primarie, 41100 Modena, Italy; 3 SIICP—Società Italiana Interdisciplinare per le Cure Primarie, 70124 Bari, Italy

**Keywords:** adult vaccination, primary care, vaccine hesitancy, social determinants, family medicine

## Abstract

Background: The number of vaccines recommended for adults and older people has increased in recent years, and same-visit coadministration represents a potential strategy to improve vaccination coverage and timeliness. However, its implementation in primary care may depend on general practitioners’ (GPs) attitudes, vaccination experience, and organisational factors. This study investigated knowledge, attitudes, and practices (KAP) regarding vaccine coadministration among GPs in Apulia, Southern Italy. Methods: We conducted an anonymous cross-sectional online survey between January and June 2026 among practicing GPs and general practice trainees working in outpatient primary care settings in Apulia. The questionnaire assessed vaccination activity, intended coadministration practices, perceived effects on vaccine effectiveness and coverage, safety in very old adults or patients with multimorbidity, and perceived barriers to vaccination. Descriptive and bivariate analyses were performed, followed by multivariable logistic regression to identify factors independently associated with a positive perception of coadministration safety in very old or multimorbid patients. An additional sensitivity analysis excluded respondents reporting insufficient information to assess safety. Results: A total of 256 physicians completed the survey; 71.1% were aged ≥50 years. Most respondents had administered adult vaccines during the previous year, and 68.0% were classified as high vaccinators, having administered three or more different vaccine types. Although 68.0% believed that coadministration could increase vaccination coverage, only 13.3% reported willingness to administer three or more vaccines during a single visit. Overall, 50.0% considered coadministration very or fairly safe in very old or multimorbid patients, 24.2% considered it slightly or not at all safe, and 25.8% reported insufficient information to assess its safety. In the fully adjusted model, high vaccination activity was associated with higher odds of a positive safety perception (adjusted odds ratio [aOR] 1.93, 95% confidence interval [CI] 1.01–3.70), whereas difficulties using vaccination IT platforms were associated with lower odds (aOR 0.48, 95% CI 0.26–0.86). Age ≥ 50 years was not independently associated with the outcome after adjustment (aOR 0.66, 95% CI 0.35–1.26). Conclusions: GPs in Apulia showed generally favorable attitudes toward vaccine coadministration, although substantial uncertainty remained regarding its use in very old and multimorbid patients. Confidence in coadministration was primarily associated with greater vaccination activity and fewer difficulties with vaccination IT platforms, whereas age-related differences observed in bivariate analyses were attenuated after multivariable adjustment. Educational, organisational, and digital-support interventions may facilitate broader implementation of same-visit adult vaccination in primary care.

## 1. Introduction

Adult immunization is a fundamental component of prevention strategies across the life course, particularly in a context of population ageing, rising multimorbidity and increasing frailty [1,2,3]. In this framework, vaccination can no longer be regarded as a purely pediatric intervention, but as an autonomous pillar of public health throughout adulthood and older age. Adult and older-adult vaccination against influenza, pneumococcal disease, COVID-19 and herpes zoster is consistently cost-effective, owing to reductions in hospitalizations, complications and long-term disability and the associated savings for health systems [4,5,6]. Nevertheless, vaccine-preventable infections continue to generate a substantial clinical and health-system burden, contributing to hospitalizations, functional decline, loss of independence and excess mortality among older and chronically ill individuals [3,4,5,6,7].

The demographic transition underway in Europe and Italy makes this issue even more urgent, as the growing proportion of older adults is accompanied by a higher prevalence of chronic diseases and reduced physiological reserve [2,3,8]. Immunosenescence, frailty and multimorbidity increase the likelihood that respiratory infections and viral reactivations will lead to severe outcomes, with important consequences for quality of life and healthcare sustainability [3,4,5,6,7]. In response to these trends, Italian National Immunization Plans since 2017 have progressively adopted a “schedule for life” approach, moving beyond an exclusive focus on children and adolescents to include structured recommendations for adults and older adults [8,9,10]. The concept of life-course immunization reflects the need to ensure appropriate vaccination opportunities not only in childhood, but also in adulthood and older age, with special attention to high-risk groups [6,7,8,9,10].

In Italy, the National Vaccination Prevention Plan 2023–2025 and the national vaccination schedule reaffirm the need to harmonize immunization strategies across the country, reduce inequities in access and promote proactive vaccination among vulnerable populations. Within this framework, vaccine coadministration represents a particularly relevant strategy, as it allows the delivery of multiple vaccines during the same visit, reduces missed opportunities for vaccination, simplifies the care pathway and improves timeliness of protection [9,11,12,13,14]. As explicitly stated in the Plan, “In the context of all vaccination offering strategies, if there are no general contraindications, it is advisable to propose co-administration”. From an organizational perspective, coadministration can benefit both patients and health services, because fewer visits mean fewer chances to postpone vaccination, greater timeliness of protection and more efficient use of resources [9,11,12]. However, adult and older-adult vaccine coverage in Italy and across Europe remains suboptimal, with large proportions of eligible individuals not receiving the recommended doses for influenza, pneumococcal disease, herpes zoster and COVID-19 [15,16,17,18].

Available evidence indicates that adult vaccine coadministration is, in most studied combinations, safe, well tolerated and immunogenic [9,14,15,16]. A recent narrative review showed that, across the combinations assessed, adverse events were generally mild to moderate and short-lived, with no consistent signals of serious toxicity or clinically meaningful impairment of immune response. Primary studies have documented the safety and immunogenicity of coadministration of pneumococcal and influenza vaccines in older adults, as well as favorable data for concomitant administration of COVID-19 and influenza vaccines in older populations. For herpes zoster vaccines and several other adult combinations, the available literature also supports an overall reassuring safety profile. Despite this favorable scientific rationale, the transition from evidence to practice is not automatic: implementation barriers continue to limit routine adoption of coadministration, generating a gap between recommendations and real-world behavior. Part of this gap is related to vaccine hesitancy, which affects not only patients but also health-care providers, who may have concerns about tolerability, effectiveness, and the potential for immune overload when multiple vaccines are administered at the same visit. Additional barriers include staffing shortages, bureaucratic complexity, information-system constraints, supply issues, and limited consultation time, all of which can hinder the practical implementation of vaccination recommendations. The literature also shows that health-care providers’ trust in vaccines and their willingness to recommend them actively are crucial determinants of vaccination uptake.

Within the community-based prevention system, general practitioners (GPs) play a central role in identifying eligible individuals, delivering vaccine counseling and translating recommendations into actual vaccination events. Their attitude toward coadministration can substantially influence both patient acceptance and the ability of the system to achieve adequate coverage in adult and frail populations [18,19,20]. In this context, Puglia—a major region in Southern Italy with a population of approximately 4 million inhabitants—represents a particularly relevant setting. The regional healthcare system is decentralized and structured across multiple Local Health Authorities (Aziende Sanitarie Locali, ASL), where GPs serve as the primary administrators of adult vaccination and are actively tasked with administering seasonal influenza, COVID-19, pneumococcal and herpes zoster vaccines to elderly and high-risk patients in their ambulatory clinics [1,20,21,22].

Assessing the perceptions and potential implementation barriers among these professionals may therefore provide valuable information for improving vaccination delivery and supporting more uniform implementation of recommendations across the territory. The present study aims to assess, through a survey among GPs in the Puglia region, their knowledge, attitudes, perceived safety, and organizational barriers regarding multiple vaccine coadministration in adults and frail individuals. By providing novel KAP data on vaccine coadministration in Italian primary care, this study seeks to identify practice- and system-level factors that can be targeted to strengthen the implementation of adult vaccination strategies in the community setting.

## 2. Materials and Methods

### 2.1. Study Design

We conducted an observational, cross-sectional knowledge, attitudes, and practices (KAP) survey using an anonymous, self-administered online questionnaire. The study aimed to investigate general practitioners’ vaccination practices, attitudes toward vaccine coadministration, perceived safety and effectiveness of coadministration, and organisational barriers to adult vaccination in primary care.

Coadministration was assessed in the questionnaire as a general vaccination strategy rather than with reference to specific vaccine combinations. Therefore, respondents were asked to express their overall views regarding the administration of multiple vaccines during the same visit, including in very old adults and patients with multimorbidity.

### 2.2. Study Setting and Participants

The survey was conducted between January and June 2026 among practicing general practitioners (GPs) and general practice trainees working in outpatient primary care settings in the Apulia region of Southern Italy.

In the Italian primary care system, GPs predominantly provide care to adult patients, whereas routine pediatric primary care is generally provided by family pediatricians. Accordingly, the present study focused specifically on vaccination in adult and older-adult populations.

Participating physicians worked in primary care settings involved in the delivery of adult vaccination within the regional implementation of national immunisation recommendations. Vaccines included in the Italian National Immunization Plan are publicly funded and provided free of charge to eligible population groups through the National Health Service. Eligible patients do not pay an additional fee for vaccination delivered within these publicly funded programmes. In Apulia, GPs contribute to the delivery of several recommended adult vaccines, including influenza, pneumococcal, herpes zoster, and SARS-CoV-2 vaccines, alongside public vaccination services.

The questionnaire was disseminated through e-mail and WhatsApp (version 2.26, Meta Platforms, Inc., Menlo Park, CA, USA) to physicians who had previously participated in educational events or seminars and/or were included in mailing lists and professional networks of the SIMCCP (Italian Society of Community Medicine and Primary Care) and SIICP (Italian Interdisciplinary Society of Primary Care). Recruitment was conducted through a Google Forms link (web-based application; Google LLC, Mountain View, CA, USA).

Participation was voluntary, and no financial or other incentives were provided. The questionnaire was completely anonymous and did not collect names, contact details, or other personal identifiers.

Because the survey link was disseminated through partially overlapping mailing lists, professional networks, educational groups, and WhatsApp channels, the exact number of unique physicians who received or viewed the invitation could not be reliably established. Consequently, a conventional survey response rate could not be calculated.

Inclusion criteria were: (1) being a practicing GP or a physician enrolled in general practice training; (2) working in an outpatient primary care setting in the Apulia region; and (3) currently providing primary care services to adult or older-adult patients.

Exclusion criteria were: (1) not working as a GP or general practice trainee, including physicians from other specialties or hospital-based physicians; (2) not having an active outpatient primary care practice in Apulia; and (3) questionnaires lacking core demographic or vaccination-activity information required for the analyses.

### 2.3. Sample Size

No formal a priori sample-size calculation was performed. All eligible GPs and general practice trainees who could be reached through the recruitment channels described above were invited to participate during the study period.

The final analytical sample consisted of all eligible respondents who completed the questionnaire and provided the information required for the principal analyses. The achieved sample size was used to provide descriptive estimates and exploratory analyses of factors associated with knowledge, attitudes, and practices regarding vaccine coadministration.

### 2.4. Questionnaire Development and Data Collection

Data were collected using a questionnaire specifically developed for the present study and administered through Google Forms.

The content and structure of the questionnaire were informed by a review of the scientific literature on adult and older-adult vaccination, vaccine coadministration, vaccine hesitancy, and vaccination delivery in primary care, together with national immunisation recommendations and relevant professional society documents.

A preliminary version of the questionnaire was pilot-tested among approximately 10 general practitioners to assess clarity, comprehensibility, completeness, and feasibility of administration in routine practice. Feedback obtained during the pilot phase was used to refine the wording and sequence of questionnaire items before dissemination of the final version.

The questionnaire collected socio-demographic and professional characteristics, including professional status, age, sex, and Local Health Authority (Azienda Sanitaria Locale, ASL Bari, Italy) affiliation. Years of professional practice were not specifically collected.

Vaccination activity was assessed by asking respondents which vaccine types they had administered in their outpatient practice during the previous year. The questionnaire also assessed the maximum number of vaccines respondents reported that they would be willing to administer during a single visit.

For analytical purposes, respondents were classified according to the number of different vaccine types administered during the previous year. High vaccinators were defined as physicians who had administered three or more different vaccine types (≥3), whereas low vaccinators were those who had administered fewer than three vaccine types (<3).

Respondents were also asked about the perceived effect of coadministration on vaccine effectiveness, whether they believed coadministration could influence vaccination coverage or patient adherence, and their perception of its safety in very old adults or patients with multimorbidity.

The questionnaire assessed coadministration as a general concept and did not separately investigate specific vaccine combinations. Accordingly, responses should be interpreted as general attitudes toward same-visit administration rather than perceptions of individual vaccine pairs.

Respondents were also asked whether, in their experience, coadministration was associated with an increase in minor adverse events, such as fever or local injection-site pain. The questionnaire did not collect individual patient-level histories of previous vaccine-related adverse events.

General perceptions of the safety of vaccines currently recommended in Italy were assessed using the response categories included in the original questionnaire, including “completely safe,” “fairly safe,” and “more long-term safety studies are needed.” These terms are reported using the wording of the original survey instrument.

The questionnaire additionally explored perceived organisational and practical barriers to vaccination, including patient concerns, vaccine supply difficulties, lack of financial incentives, shortage of nursing staff, and difficulties using vaccination IT platforms.

For the purposes of the present analysis, vaccination IT platforms referred broadly to digital platforms used by physicians in routine vaccination-related activities, particularly for the management and recording of vaccination information. Respondents were classified according to whether they reported difficulties using these platforms.

### 2.5. Data Management

Survey responses were automatically recorded through Google Forms (web based application; Google LLC, Mountain View, CA, USA) and subsequently exported to a Microsoft Excel (Microsoft 365; Microsoft Corporation, Redmond, WA, USA) spreadsheet, which served as the primary database for data cleaning and management.

Data were checked for completeness, internal consistency, and plausibility before analysis. Missing responses were handled using a complete-case approach for each individual analysis; missing observations were therefore excluded from the denominator of the corresponding variable rather than being imputed.

Statistical analyses were performed using RStudio Desktop (Open Source Edition versione 2025.09; Posit Software, PBC, Boston, MA, USA).

### 2.6. Ethical Considerations

According to the policy of the University of Bari “Aldo Moro”, anonymous surveys involving healthcare professionals that do not involve patients, clinical interventions, or identifiable personal data are exempt from formal ethics committee review.

The study was therefore conducted without formal ethics committee approval, in accordance with applicable institutional requirements and the principles of the Declaration of Helsinki.

Participation was entirely voluntary. Participants were informed about the purpose of the study and the anonymous nature of data collection. Completion and submission of the questionnaire were considered to constitute implied informed consent. No identifiable personal data were collected.

### 2.7. Statistical Analysis

Descriptive statistics were used to summarise respondents’ socio-demographic and professional characteristics, vaccination activity, intended coadministration practices, attitudes toward vaccine coadministration, and perceived barriers.

Categorical variables were presented as absolute frequencies and percentages. Unless otherwise specified, percentages were calculated using the number of available responses for each variable, with missing values excluded from the corresponding denominator.

Bivariate analyses were performed for the principal subgroups of interest, including age (<50 vs. ≥50 years), sex, vaccination activity (high vs. low vaccinators), and reported difficulties using vaccination IT platforms (yes vs. no).

Associations between categorical variables were assessed using Pearson’s chi-square test on r × c contingency tables. Fisher’s exact test was used when expected cell frequencies were <5. All statistical tests were two-sided, and a *p*-value < 0.05 was considered statistically significant.

To identify factors independently associated with confidence in vaccine coadministration among clinically vulnerable patients, a multivariable logistic regression analysis was performed.

The primary dependent variable was a positive perception of the safety of coadministration in very old adults or patients with multimorbidity, defined as respondents selecting “very safe” or “fairly safe”. Responses indicating that coadministration was “slightly safe”, “not safe at all”, or that the respondent had insufficient information to assess safety were classified as non-positive or uncertain perceptions in the primary model.

The multivariable model included age (<50 vs. ≥50 years), sex (female vs. male), vaccination activity (high vaccinator [≥3 vaccine types] vs. low vaccinator [<3 vaccine types]), reported difficulties using vaccination IT platforms (yes vs. no), professional status (general practice trainee vs. practicing GP), and ASL affiliation.

ASL affiliation was entered as a categorical variable, with ASL Bari used as the reference category.

Because only three respondents identified as non-binary, this subgroup was too small to provide stable regression estimates and was therefore excluded from analyses in which sex was included as a binary covariate.

Adjusted odds ratios (aORs), 95% confidence intervals (CIs), and *p*-values were calculated for variables included in the multivariable model. For the multi-category ASL variable, an overall test of association was also performed.

To distinguish an explicitly negative safety perception from uncertainty due to insufficient information, an additional sensitivity analysis was performed. Respondents who selected “insufficient information to assess” were excluded, and the fully adjusted logistic regression model was repeated using the same covariates. In this analysis, respondents reporting a positive safety perception (“very safe” or “fairly safe”) were compared exclusively with respondents reporting a negative safety perception (“slightly safe” or “not safe at all”).

This sensitivity analysis was used to assess whether the direction and magnitude of the associations observed in the primary model were robust when respondents expressing uncertainty were analysed separately from those expressing an explicitly negative perception.

## 3. Results

A total of 256 physicians completed the questionnaire. Most respondents were practicing general practitioners (236/256; 92.2%), while 20/256 (7.8%) were general practice trainees. The largest age group comprised physicians aged ≥60 years (134/256; 52.3%), followed by those aged 50–59 years (48/256; 18.8%), 40–49 years (38/256; 14.8%), and 27–39 years (36/256; 14.1%). The sample included 138 women (53.9%), 115 men (44.9%), and 3 respondents identifying as non-binary (1.2%) (Table 1).

Almost all respondents reported having administered vaccines in their outpatient practice during the previous year. Only 10/256 physicians (3.9%) reported administering no vaccines. A total of 91/256 (35.5%) had administered four or more different vaccine types, 83/256 (32.4%) had administered three types, 52/256 (20.3%) two types, and 20/256 (7.8%) one type. Overall, 174/256 respondents (68.0%) had administered three or more vaccine types and were therefore classified as high vaccinators, whereas 82/256 (32.0%) had administered fewer than three vaccine types and were classified as low vaccinators. The most frequently administered vaccines were influenza (95.6%), pneumococcal (88.9%), herpes zoster (69.8%), and SARS-CoV-2 vaccines (31.7%) (Table 1).

When asked about the maximum number of vaccines they would be willing to administer during a single visit, 143/256 respondents (55.9%) selected two vaccines, 79/256 (30.9%) selected one vaccine, and 34/256 (13.3%) selected three or more vaccines. Regarding the perceived effect of coadministration on vaccine effectiveness, 166/256 physicians (64.8%) believed that effectiveness would remain unchanged, whereas 44/256 (17.2%) believed that it would increase and 46/256 (18.0%) that it would decrease (Table 1).

When specifically asked about coadministration in very old adults or patients with multimorbidity, 128/256 respondents (50.0%) considered it very or fairly safe. In contrast, 62/256 (24.2%) considered it slightly or not at all safe, while 66/256 (25.8%) reported that they did not have sufficient information to assess its safety.

Regarding the safety of vaccines currently recommended in Italy, 120/255 respondents (47.1%) selected the response category “completely safe” and 111/255 (43.5%) selected “fairly safe”, whereas 24/255 (9.4%) indicated that further long-term safety studies were needed. One response was missing for this item.

Most respondents (174/256; 68.0%) believed that coadministration could increase vaccination coverage, either partially or substantially. Conversely, 43/256 (16.8%) expected no meaningful effect on coverage, and 39/256 (15.2%) believed that coadministration might discourage vaccination uptake.

The most frequently reported barrier to vaccination in primary care was patient concern or hesitancy (65.1%), followed by difficulties in vaccine supply from the Local Health Authority (37.2%), lack of financial incentives (32.4%), difficulties using vaccination IT platforms (31.2%), and shortage of nursing staff (30.1%) (Table 1). Regarding short-term reactogenicity, 161/256 respondents (62.9%) reported that, in their experience, coadministration did not increase the frequency of minor adverse events such as fever or local pain, whereas 95/256 (37.1%) reported having observed an increase.

Age-stratified analyses showed significant differences between physicians aged <50 years (*n* = 74) and those aged ≥50 years (*n* = 182) (Table 2). Physicians aged <50 years more frequently reported willingness to administer two vaccines during a single visit than physicians aged ≥50 years (67.6% vs. 51.1%), whereas the latter more frequently selected one vaccine per visit (35.2% vs. 20.3%) (χ^2^ = 6.44; *p* = 0.040). Perceived safety of coadministration in very old or multimorbid patients also differed significantly between age groups (χ^2^ = 7.70; *p* = 0.021). Physicians aged <50 years more frequently considered coadministration very or fairly safe (65.2% vs. 45.6%), whereas physicians aged ≥50 years more frequently considered it slightly or not safe (27.5% vs. 17.4%) or reported insufficient information to assess its safety (26.9% vs. 17.4%). No statistically significant age-related differences were observed in perceived vaccine effectiveness, barriers related to financial incentives, vaccination IT platforms or nursing shortages, or perceived safety of vaccines currently recommended in Italy.

No statistically significant differences were observed between women (*n* = 138) and men (*n* = 115) in the main bivariate comparisons. Willingness to administer two vaccines during a single visit was somewhat more frequent among women than men (59.4% vs. 51.3%), although the difference did not reach statistical significance (*p* = 0.075). Similarly, the proportion considering coadministration very or fairly safe in very old or multimorbid patients was 45.7% among women and 53.9% among men (*p* = 0.107).

Marked differences were observed according to vaccination activity. Of the 256 respondents, 174 (68.0%) were classified as high vaccinators (≥3 vaccine types administered during the previous year) and 82 (32.0%) as low vaccinators (<3 vaccine types). High vaccinators were more likely to report willingness to administer two vaccines during a single visit and less likely to restrict administration to one vaccine. They were also more likely to believe that coadministration does not alter vaccine effectiveness (71.8% vs. 50.0%) and less likely to believe that it reduces effectiveness (11.5% vs. 31.7%) (χ^2^ = 16.86; *p* < 0.001). A positive perception of safety in very old or multimorbid patients was also more frequent among high vaccinators than among low vaccinators (56.9% vs. 35.4%; χ^2^ = 14.41; *p* < 0.001). High vaccinators were less likely to identify lack of financial incentives as a barrier (25.3% vs. 47.6%; χ^2^ = 11.62; *p* < 0.001). Perceptions of the safety of vaccines currently recommended in Italy also differed between the two groups: 59.5% of high vaccinators selected the response category “completely safe”, compared with 20.7% of low vaccinators, whereas 24.4% of low vaccinators indicated that further long-term safety studies were needed, compared with 2.3% of high vaccinators (χ^2^ = 50.19; *p* < 0.001) (Table 3).

Overall, 179/256 physicians (69.9%) reported no difficulties using vaccination IT platforms, whereas 77/256 (30.1%) reported such difficulties. Vaccination activity did not differ significantly between these groups, although physicians without IT difficulties more frequently reported administering three or more vaccine types than those with IT difficulties (72.6% vs. 57.1%; χ^2^ = 5.93; *p* = 0.052). Similarly, the maximum number of vaccines respondents were willing to administer during a single visit did not differ significantly according to IT difficulties (χ^2^ = 4.57; *p* = 0.102), although physicians without IT difficulties more frequently selected two vaccines (59.2% vs. 48.1%) and less frequently selected one vaccine (26.8% vs. 40.3%) (Table 4).

More substantial differences were observed in perceptions of vaccine effectiveness and safety. Physicians without IT difficulties more frequently believed that coadministration does not alter vaccine effectiveness (69.3% vs. 54.5%; χ^2^ = 10.65; *p* = 0.005), whereas the belief that effectiveness decreases was more frequent among physicians reporting IT difficulties (29.9% vs. 12.8%). Perceived safety in very old or multimorbid patients also differed markedly between groups: 56.4% of physicians without IT difficulties considered coadministration very or fairly safe, compared with 31.0% of those reporting IT difficulties (χ^2^ = 15.50; *p* < 0.001). Conversely, 37.9% of physicians with IT difficulties considered coadministration slightly or not safe, compared with 21.8% of those without IT difficulties. Perceptions of vaccines currently recommended in Italy also differed significantly, with 53.4% of physicians without IT difficulties selecting the response category “completely safe”, compared with 32.5% of physicians reporting IT difficulties (χ^2^ = 9.56; *p* = 0.008) (Table 4).

In the multivariable logistic regression analysis, after adjustment for age, sex, vaccination activity, difficulties using vaccination IT platforms, professional status, and Local Health Authority (ASL) affiliation, high vaccination activity remained independently associated with a positive perception of the safety of vaccine coadministration in very old adults or patients with multimorbidity. Physicians who had administered three or more vaccine types during the previous year had higher odds of reporting a positive safety perception than low vaccinators (aOR 1.93, 95% CI 1.01–3.70; *p* = 0.048). Conversely, physicians reporting difficulties using vaccination IT platforms had lower odds of a positive safety perception (aOR 0.48, 95% CI 0.26–0.86; *p* = 0.014) (Table 5).

The association observed for age in the bivariate analysis was attenuated after multivariable adjustment and was no longer statistically significant (age ≥50 years vs. <50 years: aOR 0.66, 95% CI 0.35–1.26; *p* = 0.210). Sex and professional status were likewise not significantly associated with the primary outcome (female vs. male: aOR 0.70, 95% CI 0.41–1.20; *p* = 0.199; general practice trainee vs. practicing GP: aOR 1.67, 95% CI 0.52–5.37; *p* = 0.389). No statistically significant overall association was observed between ASL affiliation and positive safety perception (global *p* = 0.361) (Table 5).

A sensitivity analysis was subsequently performed to distinguish an explicitly negative safety perception from uncertainty due to insufficient information. Respondents who selected “I do not have sufficient information to assess” were excluded, and the fully adjusted model was repeated, comparing respondents who considered coadministration very or fairly safe with those who considered it slightly or not at all safe. In this analysis, difficulties using vaccination IT platforms remained significantly associated with lower odds of a positive safety perception (aOR 0.43, 95% CI 0.20–0.93; *p* = 0.031). High vaccination activity remained associated with higher odds of a positive safety perception, with a similar magnitude of association, although the confidence interval marginally included the null value (aOR 2.14, 95% CI 0.98–4.65; *p* = 0.055). Age remained non-significantly associated with the outcome (aOR 0.52, 95% CI 0.22–1.25; *p* = 0.145). Female sex was associated with lower odds of a positive safety perception in this sensitivity analysis (aOR 0.47, 95% CI 0.23–0.95; *p* = 0.036), whereas professional status and ASL affiliation showed no significant overall associations with the outcome (Table S1).

## 4. Discussion

This survey among general practitioners in Apulia provides a detailed picture of how vaccine coadministration is perceived and potentially implemented in primary care. Overall, the findings highlight a gap between routine adult vaccination activity and full acceptance of same-visit administration of multiple vaccines. Although most respondents had administered several different vaccine types during the previous year, only a minority reported that they would be willing to administer three or more vaccines during the same visit [23,24].

Respondents generally expressed a favorable, although not uniformly confident, attitude toward coadministration. Most believed that coadministration does not reduce vaccine effectiveness and that it could improve vaccination coverage. These findings are consistent with previous evidence showing that clinicians and other stakeholders recognize the practical advantages of administering multiple vaccines during the same visit, particularly by reducing missed opportunities and the number of healthcare encounters required [23,24,25,26]. A recent qualitative scoping review of adult vaccine coadministration similarly reported that coadministration is generally perceived as convenient and potentially beneficial for vaccine uptake, while concerns regarding safety, reactogenicity, and the burden of receiving multiple injections remain relevant [24]. Studies among pediatricians have also shown that healthcare professionals may appreciate strategies that reduce the number of visits while simultaneously expressing concerns regarding safety and immunogenicity when several antigens are administered together [27]. Taken together, these findings suggest that support for coadministration can coexist with residual uncertainty regarding its practical implementation.

Age-related differences were evident in the bivariate analyses. Physicians aged <50 years more frequently reported willingness to administer two vaccines during a single visit and were more likely to consider coadministration safe in very old or multimorbid patients. Conversely, physicians aged ≥50 years more frequently selected one vaccine per visit and more often expressed either a negative safety perception or insufficient information to assess safety. Previous studies have suggested that age and professional generation may be associated with differences in attitudes toward vaccination, adoption of new practices, and familiarity with evolving healthcare technologies [24,28,29]. However, an important finding of the revised multivariable analysis is that the association with age was attenuated after adjustment for vaccination activity, IT difficulties, professional status, and Local Health Authority affiliation and was no longer statistically significant. Therefore, age itself should not be interpreted as an independent determinant of confidence in coadministration. The differences observed in the bivariate analysis may instead partly reflect variation in vaccination experience, exposure to newer recommendations, professional context, or familiarity with digital and organisational processes.

Vaccination activity represented one of the clearest signals in the study. Physicians who had administered three or more different vaccine types during the previous year were more likely to consider coadministration safe, more likely to believe that it does not reduce vaccine effectiveness, and less likely to identify lack of financial incentives as a barrier. In the fully adjusted model, high vaccination activity remained associated with approximately twice the odds of reporting a positive safety perception compared with lower vaccination activity. This finding is consistent with the hypothesis that greater practical familiarity with vaccination delivery may be associated with greater confidence in coadministration [23,27,30].

This association should nevertheless be interpreted cautiously. Because of the cross-sectional design, the direction of the relationship cannot be established. Physicians who routinely administer a broader range of vaccines may become more familiar and comfortable with vaccination and coadministration over time; alternatively, physicians who already have greater confidence in vaccination may be more likely to offer a broader range of vaccines in their practice. Thus, rather than demonstrating a causal “implementation culture”, the present findings suggest a close relationship between vaccination practice and confidence that may operate in both directions. In the sensitivity analysis excluding respondents who reported insufficient information to assess safety, the magnitude of this association remained similar, although the confidence interval marginally crossed unity, likely also reflecting the reduced analytical sample.

Difficulties using vaccination IT platforms were another consistent finding. Physicians reporting such difficulties were less likely to consider coadministration safe in very old or multimorbid patients and more frequently expressed concerns regarding vaccine effectiveness and safety. Importantly, the association between IT difficulties and lower odds of a positive safety perception remained statistically significant both in the fully adjusted primary model and in the sensitivity analysis. A 2023 systematic review of digital health adoption among healthcare professionals identified technical and infrastructural limitations, perceived complexity, psychological barriers, and workload concerns as recurrent obstacles to the implementation of digital health technologies [31]. More recent evidence similarly indicates that organisational and usability barriers remain common across healthcare professions [32], while difficulties with digital technologies may interact with digital literacy, perceived complexity, and confidence in new systems [33].

Our findings do not demonstrate that difficulties with IT platforms directly cause lower confidence in vaccination or coadministration. Rather, such difficulties may represent a marker of a more complex operational environment in which administrative burden, technological friction, and vaccination delivery intersect. Physicians experiencing greater difficulty with vaccination-related digital systems may have less time or organisational capacity to incorporate additional preventive interventions during routine visits. Conversely, greater familiarity with vaccination activity itself may also increase familiarity with the associated digital tools. The cross-sectional nature of the study does not allow these pathways to be disentangled.

The inclusion of professional status and Local Health Authority affiliation in the revised multivariable model provides additional information regarding the organisational context. Neither being a general practice trainee rather than a practicing GP nor ASL affiliation showed a statistically significant overall association with the primary outcome. This suggests that the observed differences in confidence cannot be readily attributed to professional status or to a specific Local Health Authority within Apulia. However, subgroup sizes were relatively small for trainees and for some individual ASLs, and the absence of statistically significant associations should therefore not be interpreted as evidence that organisational context is irrelevant. Larger studies specifically designed to investigate between-area variation may be better suited to examining this issue.

The practical barriers reported by respondents also deserve attention. Patient concerns or hesitancy were the most frequently reported obstacles, followed by vaccine supply difficulties, lack of financial incentives, difficulties with IT platforms, and shortage of nursing staff. This pattern is consistent with broader models of vaccine hesitancy and uptake, in which confidence, convenience, and contextual barriers interact in determining whether vaccination is ultimately delivered [34]. In primary care, these factors are unlikely to operate independently. Even when a physician is willing to recommend and administer several vaccines, patient hesitancy, lack of vaccine availability, administrative complexity, or insufficient staff may prevent same-visit vaccination. This may help explain why most respondents believed that coadministration could improve vaccination coverage while a substantial minority remained cautious about its routine implementation.

The finding that 37.1% of respondents reported an increase in minor adverse events with coadministration also requires nuanced interpretation. Evidence generally supports the safety and immunogenicity of adult vaccine coadministration, including in older populations, with most reported adverse events being mild and transient [9,11,12]. However, this does not imply that physicians reporting greater short-term reactogenicity are necessarily misinterpreting clinical experience. When two or more vaccines are administered during the same visit, patients may experience local reactions at more than one injection site or several transient symptoms during the same post-vaccination period. Consequently, the burden of minor symptoms experienced during a single vaccination episode may be perceived as greater even when coadministration does not increase serious adverse events or compromise the overall safety profile. Short-term reactogenicity should therefore be regarded as a legitimate practical consideration that may influence both physician recommendations and patient acceptance.

An additional consideration is that the questionnaire assessed coadministration as a general concept rather than distinguishing specific vaccine combinations. This is relevant because reactogenicity, immunogenicity, and perceived acceptability may differ across combinations, including influenza–pneumococcal vaccination, influenza–COVID-19 vaccination, and combinations involving recombinant herpes zoster vaccine [9,14,15,16]. Accordingly, the present results should be interpreted as reflecting physicians’ general attitudes toward same-visit administration rather than their assessment of the safety or acceptability of any individual vaccine pair. Future studies should examine specific combinations separately and investigate whether physicians’ willingness to coadminister varies according to vaccine type, platform, or expected reactogenicity.

One of the strengths of the present study is its focus on the Italian primary care setting. Adult vaccine coadministration among GPs remains relatively under-explored in Italy, despite the increasingly important role of primary care in life-course immunisation. Most respondents in our sample were already involved in administering several adult vaccines, indicating that the primary care network provides an established platform on which broader coadministration strategies could build. At the same time, the relatively low proportion of physicians willing to administer three or more vaccines during a single visit suggests that formal recommendations alone may not be sufficient to ensure widespread implementation.

A previous cross-sectional study conducted among 335 general practitioners in Piedmont, Northern Italy, also reported generally favorable attitudes toward adult vaccination. In that study, 68.1% of respondents reported planning to coadminister vaccines, although 55.5% indicated a need for further information on vaccine coadministration [35]. Younger GPs were also more comfortable proposing some adult vaccines, particularly Tdap and herpes zoster vaccination. Although the questionnaire and outcomes were not directly comparable with those used in the present study, these findings suggest that both Northern and Southern Italian GPs generally recognize the value of adult vaccination while retaining educational needs and some uncertainty regarding coadministration.

Direct geographical comparisons should nevertheless be made cautiously. The present study was conducted exclusively in Apulia and was not designed to assess differences between Northern and Southern Italy. Regional organisation of vaccination services, involvement of GPs, digital infrastructure, and local implementation strategies may differ across the country. Future multicentre studies involving both Northern and Southern Italian regions would therefore be useful to determine whether the patterns observed in Apulia are shared nationally or reflect specific regional characteristics.

From an implementation perspective, the revised findings suggest two particularly relevant areas for intervention. First, greater practical familiarity with adult vaccination was consistently associated with greater confidence in coadministration, supporting educational and organisational strategies that facilitate routine vaccine delivery in primary care. Second, difficulties with vaccination IT platforms remained independently associated with lower confidence even after adjustment for demographic, professional, and organisational variables. Simplifying vaccination-related digital processes and ensuring adequate technical support may therefore complement educational interventions. In contrast, the age-related differences observed in descriptive analyses should not be used alone to identify physicians requiring support, as age was not independently associated with safety perception in the fully adjusted model.

Several limitations should be considered when interpreting these findings. First, participants were recruited through professional networks, educational activities, scientific society mailing lists, and WhatsApp channels. This recruitment strategy may have preferentially reached physicians with greater interest in continuing education or vaccination and may therefore limit the generalisability of the findings. Because these recruitment channels partially overlapped and the survey link could circulate within professional networks, the exact number of unique physicians who received or viewed the invitation could not be established; consequently, a conventional response rate could not be calculated.

Second, the questionnaire was self-administered and relied on self-reported practices and perceptions. Responses may therefore have been affected by recall bias and social desirability bias, and reported willingness to coadminister vaccines should not necessarily be interpreted as directly observed clinical behaviour.

Third, as noted above, coadministration was assessed as a general concept rather than for specific vaccine combinations. Differences in reactogenicity, evidence base, and clinical familiarity between vaccine pairs could therefore not be explored. Future versions of the questionnaire should include combination-specific items.

Fourth, when assessing the general safety of vaccines currently recommended in Italy, the questionnaire included the response category “completely safe”. No medical intervention is entirely risk-free, and this absolute wording may have influenced respondents’ interpretation of the item. Some physicians may have selected a more cautious response despite holding an overall favourable view of vaccine safety. Future versions of the survey should therefore use more nuanced response categories.

Fifth, years of professional practice were not collected. Although age may partly reflect career stage, age and professional experience are not equivalent, and years in clinical practice could independently influence familiarity with vaccination strategies. Similarly, the questionnaire did not systematically collect physicians’ previous clinical experiences with vaccine-related adverse events among their patients, which could have influenced perceptions of safety and reactogenicity.

Sixth, the original binary regression outcome combined respondents expressing an explicitly negative safety perception with those reporting insufficient information to judge. Because these represent conceptually different cognitive states, an additional sensitivity analysis was performed excluding the uncertain group. The principal association with IT difficulties remained significant, while the association with high vaccination activity remained similar in magnitude but became marginally non-significant. This supports the overall consistency of the findings while also highlighting the importance of distinguishing uncertainty from negative attitudes in future studies.

Finally, the cross-sectional design prevents causal inference. Associations between vaccination activity, digital barriers, and confidence may be bidirectional or influenced by unmeasured factors. Subgroup and multivariable analyses should therefore be interpreted as identifying associations rather than determinants or causal effects.

Despite these limitations, the study has several strengths. It includes a substantial sample of physicians working within a defined regional primary care system, examines both reported vaccination activity and attitudes, and evaluates practical as well as organisational barriers. The revised multivariable analysis additionally accounts for professional status and Local Health Authority affiliation, while the sensitivity analysis addresses uncertainty in the measurement of the primary outcome. Taken together, the findings suggest that practical vaccination experience and digital accessibility may be particularly relevant correlates of confidence in adult vaccine coadministration and may help inform future educational and organisational strategies in primary care.

## 5. Conclusions

In conclusion, our results show that general practitioners in Puglia have sufficient confidence in vaccine co-administration during the same visit, but this confidence is still incomplete and strongly depends on age, vaccination experience, and practical obstacles such as digital difficulties.

The main take-home message is that coadministration is seen as useful and potentially effective, but its wider adoption will require not only evidence but also simpler workflows, targeted training, and support for physicians who are less familiar or less comfortable with it. In addition, the number of vaccines targeting adult and older adult populations is expected to increase in the coming years, as highlighted by recent life-course immunization reports and vaccine pipeline reviews showing that a large share of new candidates is designed for adult use [8]. In this evolving context, same-visit coadministration is likely to become an increasingly important tool to deliver timely protection, reduce missed opportunities, and sustain coverage in ageing populations.

In practical terms, this means that improving vaccination coverage is not only a matter of having the right recommendations on paper. It also means making coadministration easy to deliver in everyday primary care, especially for older and frailer patients who benefit most from reducing missed opportunities.

A clear public health strategy should therefore combine scientific reassurance, organizational support, and digital simplification. If these conditions are met, coadministration could become a more routine and effective tool to strengthen adult immunization in the community setting.

## Figures and Tables

**Table 1:** Main characteristics of the sample and overall survey results.

Domain	Variable	Category	*n*	%
Participant characteristics	Professional status	Practicing general practitioner	236	92.2
		General practice trainee	20	7.8
	Age	27–39 years	36	14.1
		40–49 years	38	14.8
		50–59 years	48	18.8
		≥60 years	134	52.3
	Sex	Female	138	53.9
		Male	115	44.9
		Non-binary	3	1.2
Vaccination activity in the previous year	Number of different vaccine types administered	None	10	3.9
		One	20	7.8
		Two	52	20.3
		Three	83	32.4
		Four	78	30.5
		Five	13	5.1
	Vaccination activity category	High vaccinator (≥3 vaccine types)	174	68.0
		Low vaccinator (<3 vaccine types)	82	32.0
	Vaccine types administered *	Influenza	245	95.6
		Pneumococcal	228	88.9
		Herpes zoster	179	69.8
		SARS-CoV-2	81	31.7
Intended coadministration practice	Maximum number of vaccines respondents would administer during a single visit	One	79	30.9
		Two	143	55.9
		Three or more	34	13.3
Perceptions of coadministration	Perceived effect on vaccine effectiveness	Increases	44	17.2
		Remains unchanged	166	64.8
		Decreases	46	18.0
	Safety in very old adults or patients with multimorbidity	Very/fairly safe	128	50.0
		Slightly/not at all safe	62	24.2
		Insufficient information to assess	66	25.8
	Perceived effect on vaccination coverage	Increases coverage	174	68.0
		No meaningful impact	43	16.8
		May reduce adherence	39	15.2
	Minor adverse events with coadministration	Increased	95	37.1
		Not increased	161	62.9
General perceptions of vaccine safety	Safety of vaccines currently recommended in Italy †	“Completely safe”	120	47.1
		“Fairly safe”	111	43.5
		More long-term safety studies needed	24	9.4
Perceived barriers to vaccination in primary care *	Main reported barriers	Patient concerns/hesitancy	167	65.1
		Vaccine supply difficulties	95	37.2
		Lack of financial incentives	83	32.4
		Difficulties using vaccination IT platforms	80	31.2
		Shortage of nursing staff	77	30.1

**Notes**: Data are presented as number (*n*) and percentage (%). Percentages were calculated using the total sample (N = 256), unless otherwise specified. * Multiple responses were possible; therefore, percentages do not sum to 100%. † One response was missing for this item (*n* = 255). The expressions “completely safe” and “fairly safe” reproduce the response categories used in the questionnaire. High vaccinators were defined as physicians who had administered ≥3 different vaccine types during the previous year; low vaccinators were those who had administered <3 vaccine types.

**Table 2:** Age-related differences in attitudes and intended practices regarding vaccine coadministration.

Variable	Age < 50 Years (%)	Age ≥ 50 Years (%)	Δ Percentage Points	*p*-Value
Maximum vaccines per visit: one	20.3	35.2	+14.9	0.040
Maximum vaccines per visit: two	67.6	51.1	−16.5	0.040
Maximum vaccines per visit: three or more	12.2	13.7	+1.5	0.040
Coadministration very/fairly safe in very old or multimorbid patients	65.2	45.6	−19.6	0.021
Coadministration slightly/not safe in very old or multimorbid patients	17.4	27.5	+10.1	0.021
Insufficient information to assess safety in very old or multimorbid patients	17.4	26.9	+9.5	0.021
Coadministration effectiveness remains unchanged	60.8	66.5	+5.7	0.205
Lack of financial incentives reported as a barrier	28.4	34.1	+5.7	0.186
Vaccines currently recommended in Italy rated “completely safe”	51.4	45.1	−6.3	0.076

**Note:** Δ indicates the percentage-point difference calculated as Age ≥ 50 years minus Age < 50 years. *p*-values refer to the overall comparison across response categories where applicable.

**Table 3:** Differences between high and low vaccinators in vaccination practice and attitudes.

Variable	High Vaccinators %	Low Vaccinators %	Δ	*p*-Value
Maximum vaccines per visit: one	24.1	45.1	21	<0.001
Maximum vaccines per visit: two	58.6	50	−8.6	<0.001
Maximum vaccines per visit: three or more	17.2	4.9	−12.3	<0.001
Efficacy decreases with coadministration	11.5	31.7	20.2	<0.001
Efficacy remains unchanged	71.8	50	−21.8	<0.001
Coadministration safe in older adults	56.9	35.4	−21.5	<0.001
Lack of financial incentives is a barrier	25.3	47.6	22.3	<0.001
Vaccines recommended in Italy: completely safe	59.5	20.7	−38.8	<0.001
More long-term safety studies are needed	2.3	24.4	22.1	<0.001

**Table 4:** IT-related barriers. Comparison of physicians with and without IT difficulties.

Variable	No IT Difficulties %	IT Difficulties %	Δ	*p*-Value
Vaccines administered in the previous year: three or more types	72.6	57.1	−15.5	0.052
Maximum vaccines per session: one	26.8	40.3	13.5	0.102
Maximum vaccines per session: two	59.2	48.1	−11.1	0.102
Maximum vaccines per session: three or more	14.0	11.7	−2.3	0.102
Coadministration efficacy unchanged	69.3	54.5	−14.8	0.005
Coadministration efficacy decreases	12.8	29.9	17.1	0.005
Coadministration safe in older/multimorbid patients	56.4	31.0	−25.4	<0.001
Coadministration not safe in older/multimorbid patients	21.8	37.9	16.1	<0.001
Vaccines recommended in Italy: completely safe	53.4	32.5	−20.9	0.008
Vaccines recommended in Italy: more long-term studies needed	7.9	13.0	5.1	0.008

**Table 5:** Multivariable logistic regression analysis of factors independently associated with a positive perception of the safety of vaccine co-administration in very old adults and patients with multimorbidity.

Variable	Comparison	Adjusted OR	95% CI	*p*-Value
Age	≥50 years vs. <50 years	0.66	0.35–1.26	0.21
Sex	Female vs. male	0.7	0.41–1.20	0.199
Vaccination activity	High vaccinator (≥3 vaccine types) vs. low vaccinator (<3 vaccine types)	1.93	1.01–3.70	0.048
Difficulties using vaccination IT platforms	Yes vs. no	0.48	0.26–0.86	0.014
Professional status	General practice trainee vs. practicing GP	1.67	0.52–5.37	0.389
Local Health Authority (ASL)	BAT vs. Bari	1.91	0.57–6.40	0.296
	Brindisi vs. Bari	0.84	0.30–2.36	0.734
	Foggia vs. Bari	0.96	0.37–2.47	0.927
	Lecce vs. Bari	0.46	0.19–1.16	0.099
	Taranto vs. Bari	0.66	0.29–1.52	0.334

Abbreviations: aOR, adjusted odds ratio; CI, confidence interval; ASL, Azienda Sanitaria Locale (Local Health Authority); GP, general practitioner. Note: The overall association between ASL affiliation and positive safety perception was not statistically significant (global *p* = 0.361). The regression analysis including sex was restricted to respondents identifying as male or female; three respondents identifying as non-binary were excluded because the subgroup was too small to provide stable regression estimates.

## Data Availability

The data that support the findings of this study are not publicly available due to privacy and ethical reasons, as they derive from an anonymous survey among healthcare professionals. Aggregated, de-identified data may be made available from the corresponding author upon reasonable request and in accordance with the policy of the University of Bari “Aldo Moro”.

## Supplementary Materials

The following supporting information can be downloaded at: https://www.mdpi.com/article/10.3390/vaccines14090725/s1, Table S1. Sensitivity analysis of factors associated with a positive perception of the safety of vaccine coadministration, excluding respondents with insufficient information to assess safety.
