# Supplementary material for: Vaccine Coadministration in Primary Care: A KAP Survey Among General Practitioners in Southern Italy
Source: Vaccines (Basel). 2026 Aug 22;14(9):725. doi: 10.3390/vaccines14090725 (PMC13611679; doi:10.3390/vaccines14090725)
Supplement: Supplementary file 1 [file vaccines-14-00725-s001.zip › vaccines-4480713-supplementary.pdf]

Table S1. Sensitivity analysis of factors associated with a positive perception of the safety of vaccine coadministration, excluding respondents with insufficient information to assess safety

Variable	Comparison	Adjusted OR	95% CI	p-value
**Age**	≥50 years vs. <50 years	0.52	0.22–1.25	0.145
**Sex**	Female vs. male	**0.47**	**0.23–0.95**	**0.036**
**Vaccination activity**	High vaccinator (≥3 vaccine types) vs. low vaccinator (<3 vaccine types)	2.14	0.98–4.65	0.055
**Difficulties using vaccination IT platforms**	Yes vs. no	**0.43**	**0.20–0.93**	**0.031**
**Professional status**	General practice trainee vs. practicing GP	1.84	0.37–9.17	0.458
**Local Health Authority (ASL)**	BAT vs. Bari	0.62	0.17–2.28	0.469

	Brindisi vs. Bari	0.61	0.17–2.12	0.434
	Foggia vs. Bari	0.48	0.15–1.53	0.213
	Lecce vs. Bari	**0.30**	**0.10–0.90**	**0.032**
	Taranto vs. Bari	0.38	0.14–1.06	0.063
